# Anti-Biofouling Performance of an Immobilized Indigenous Quorum Quenching Bacterium *Bacillus cereus* HG10 and Its Influence on the Microbial Community in a Bioreactor

**DOI:** 10.3390/ijerph16193777

**Published:** 2019-10-08

**Authors:** Fangfang Xu, Chang Zhao, Chuang Hak Lee, Wenzhao Wang, Qiyong Xu

**Affiliations:** 1 College of Life Sciences and Oceanography, Shenzhen University, Shenzhen 518055, China; ff.xu@szu.edu.cn; 2 Shenzhen Engineering Laboratory for Recycled Eco-efficient Materials, School of Environment and Energy, Peking University Shenzhen Graduate School, Xili University Town, Shenzhen 518055, China; zhaochangge@126.com; 3 Fairylands Environmental Sci-Tech (Shenzhen) Crop. Ltd., Shenzhen 518055, China; leech@snu.ac.kr

**Keywords:** *Bacillus cereus*, biofouling, quorum quenching, 16S rRNA gene profiling

## Abstract

Quorum quenching-membrane bioreactors (QQ-MBRs) have been studied widely in recent decades. However, limited information is known about the influence of QQ on the microbial community. In this study, the indigenous QQ bacterium *Bacillus cereus* HG10 was immobilized and used to control biofouling in a bioreactor. QQ beads caused extracellular polymeric substance reduction and significantly hindered biofilm formation on a submerged membrane. Community profiling of 16S rRNA gene amplicons revealed that QQ beads dramatically altered the bacterial community structure in activated sludge but not in biofilm. Bacterial structure in the presence of QQ beads showed a clear divergence from that of the control groups at phylum, class, order, family, and genus taxonomic ranks. A significant enrichment of several bacterial genera, including *Acinetobacter*, *Aeromonas*, *Delftia*, *Bacillus*, and *Pseudomonas*, and depletion of over 12 bacterial genera were observed. These findings would contribute to a better understanding of why and how immobilized QQ bacteria impair membrane biofouling in QQ-MBRs.

## 1. Introduction

Membrane bioreactors (MBRs) have drawn substantial attention worldwide as wastewater treatment technology. MBRs outcompete other technologies because of several advantages, including smaller footprints, better effluent quality, and less sludge production. However, membrane biofouling, a critical obstacle, hindered the widespread use of the technology and remains to be overcome [1,2,3]. Membrane biofouling leads to lower permeate flux, higher energy consumption and cost, and it requires frequent membrane cleaning and replacement. To date, diverse anti-biofouling strategies have been reported, including the modification of membrane materials, the optimization of operating parameters, and the addition of chemicals [3,4,5,6,7]. Biofilm formation is known to be controlled and regulated by bacterial cell-to-cell communication via signal molecules called quorum sensing (QS). In recent years, quorum quenching (QQ, inhibition of cell-to-cell communication) has emerged as an innovative approach for biofouling control in an MBR [6,8,9,10]. QQ alleviates biofilm formation by degrading or modifying QS signal molecules. This is because QQ is a valuable, low cost, and sustainable method, and it is a promising approach to hinder biofouling. 

While activated sludge in MBRs consists of diverse microbiota, biofilm formation on the membrane is known to be regulated by QS [11,12]. Several microbial species in activated sludge have been reported to produce different types of signal molecules, including N-acylhomoserine lactones (AHL/AI-1) [13,14,15], autoinducer-2 (AI-2) [16,17].

Although there is broad interest to interrupt bacterial QS by QQ bacteria, limited attempts have been made to disclose the mechanism and influence of QQ bacteria on the microbial community inside bioreactors. Microbial community profiling by 16S ribosomal RNA (rRNA) gene analysis may offer valuable information to determine the impact of QQ bacteria on the bacterial community structure and unveil the relationship between QS and QQ bacteria. In this study, QQ beads were prepared by immobilizing an indigenous QQ bacterium, *Bacillus cereus* HG10, isolated from activated sludge, in sodium alginate beads. The QQ performance of the HG10 beads was then assessed in a batch bioreactor. Furthermore, microbial community analysis in both activated sludge and biofilm formed on the membrane in a batch bioreactor was conducted using high-throughput 16S rRNA barcoding and sequencing.

## 2. Materials and Methods 

### 2.1. Chemicals

Flat-sheet Durapore polyvinyl difluoride (PVDF) membrane (GVWP04700, 0.22 µm, 47 mm) was purchased from Merck Millipore (USA). Sodium alginate was purchased from Sigma (USA). Luria-Bertani (LB), agar, yeast extract and tryptone were purchased from HKM (China). Glutaraldehyde and anhydrous ethanol were purchased from Xilong Science (China). SYTO9 dye was purchased from Molecular Probes (USA). The PowerSoil® DNA Isolation Kit was purchased from MOBIO (MoBio, Carlsbad, CA, USA). Agarose was purchased from Biowest (Spain). The 2x Premix Taq DNA polymerase (Cat. #R004A) was purchased from TAKARA (Takara Bio, Terra Bella Ave. Mountain View, CA, USA). Universal primers 27F (5’-AGAGTTTGATCCTGGCTCAG-3’) and 1492R (5’-GGCTACCTTGTTACGACTT-3’) were synthesized by the Beijing Genomics Institute (BGI, Shenzhen).

### 2.2. Synthetic Wastewater

Each liter of synthetic wastewater (pH 7.0–7.5) constituted 1600 mg glucose, 56 mg yeast extract, 460 mg tryptone, 340 mg NH_4_Cl, 87 mg KH_2_PO_4_, 9.8 mg CaCl_2_, 128 mg MgSO_4_.7H_2_O, 9.44 mg MnCl_2_.4H_2_O, 8 mg FeCl_3_.6H_2_O, and 1022 mg NaHCO_3_ [15]. Sterilized (121 °C, 15 min autoclave) double distilled water was used to prepare the synthetic wastewater. The wastewater was prepared fresh each day.

### 2.3. Bead Preparation

QQ bacteria were isolated and screened from activated sludge in a wastewater treatment plant (Nanshan, Shenzhen) according to Ochiai et al. [18]. The AHL degrading capacity of the isolates was measured as described by Zhao et al. [19]. *Bacillus cereus* HG10 (CCTCC AB 2018234, China Center for Type Culture Collection), the strain with the highest AHL-degrading activity, was chosen as the candidate for anti-biofouling and microbial community shift evaluation in this research.

Both vacant and HG10-entrapped beads were prepared as described by Kim et al. [10]. Briefly, an overnight culture of HG10 was harvested, washed, and resuspended in ultrapure water to a concentration of 100 mg dry weight bacteria per mL. Then, 1 mL of bacterial suspension and 9 mL of 4% (W/V) sodium alginate were vigorously mixed and added to 3% (W/V) CaCl_2_. The mixture was incubated at 4 °C for 12 h to allow full crosslinking of the chemicals. Vacant beads were prepared following the same procedure without the addition of bacteria. The characteristics of the beads were then examined by scanning electron microscopy (SEM, ZEISS SUPRA®55).

### 2.4. Operation of the Batch Bioreactor 

A conical flask (150 mL) was chosen to simulate a batch bioreactor. Sixty conical flasks were divided into three groups (QQ beads—QQ, vacant beads—VB, and no beads control—CT) and operated in parallel. Forty-nine milliliters of synthetic wastewater and 1 mL of acclimated activated sludge (Mixed liquor suspended solids, MLSS 10 g/L) together with a sheet of PVDF membrane were added to each batch bioreactor. Activated sludge was mixed well with wastewater during the entire growth period with shaking. Finally, 15 HG10 entrapping beads or vacant beads were added to the QQ and VB groups, respectively. The bioreactors were then incubated with shaking at 30 °C, 70 r/min. A 48-h adaption time was allowed for the adaption of entrapped QQ bacteria to the system. At each 24 h time point, 25 mL of the synthetic wastewater in each batch bioreactor was replaced with freshly prepared wastewater. The biofilm formation process and biofouling conditions of each membrane were observed and recorded in all samples operated in parallel. Activated sludge and membrane samples were taken and analyzed on 1, 8, and 20 days respectively.

### 2.5. Analytical Methods

American Public Health Association, APHA 5520 C closed reflux titrimetric method [20] was used to measure chemical oxygen demand (COD) in the present study.

Extracellular polymeric substances (EPS) were extracted from the membrane by the heat extraction method [10] on the eighth day. The polysaccharides in EPS were quantified using the Lowry method [21]. Proteins in the EPS were quantified by the phenol-sulfuric acid method [22].

### 2.6. SEM Observation of Biofouling

Structures of HG-10 entrapping and vacant beads were observed by scanning electron microscopy (SEM, ZEISS SUPRA®55, Berlin, Germany). The beads were washed three times using deionized water and then fixed with 2.5% (V/V) glutaraldehyde at 4 °C for 2 h. The fixative was then washed off. The sample was then dehydrated using a graded series of ethanol (30%, 50%, 70%, 80%, 90%, and 95%) for 15 min each and finalized with 100% ethanol for 20 min. Finally, the samples were freeze-dried, and the cross sections of the beads were cut and sputtered with gold for 15 s. Then, the cross sections of beads were visualized and characterized with 1000, 2000, and 6000× magnification

Meanwhile, biofilms on the membranes at the initial (first day) and relatively mature stage (eighth day) were also observed using SEM. A piece (1 × 1 cm) of the membrane fiber was cut and observed by SEM at 1000, 5000, and 10,000× magnification. 

### 2.7. Sample and 16S Library Preparation

Three milliliters of water samples were collected on days 1, 11, and 20 from each group. Samples were frozen at −80 °C immediately after collection. Meanwhile, biofilm samples were collected by cutting a piece of each membrane sample (1 × 1 cm). The membrane pieces were then ultrasound-treated for 3–5 min with 3 mL of ddH_2_O. The microbe suspension was then taken and stored at −80 °C for 16S rRNA gene sequencing. DNA extraction was performed using a MO BIO PowerSoil® DNA Isolation Kit. DNA quality was monitored by 1% agarose gel electrophoresis.

The V3–V5 regions of 16S rRNA genes were amplified using primers 515F and 806R. PCR was carried out in a BioRad S1000 (Bio-Rad Laboratory, Foster, CA, USA). PCR amplification was carried out as follows: 94 °C for 5 min, followed by 30 cycles each of 94 °C for 30 s, 52 °C for 30 s, and 72 °C for 30 s and a final extension at 72 °C for 10 min. Amplicons were generated (NEBNext® Ultra™ DNA Library Prep Kit for Illumina®, New England Biolabs, MA, USA), quality controlled (Qubit@ 2.0 Fluorometer, Thermo Fisher Scientific, Waltham, MA, USA and Agilent Bioanalyzer 2100 system, Agilent Technologies, Waldbron, Germany), then sequenced (IlluminaHiseq2500).

All microorganisms or microbial communities mentioned in this research refer to the bacterial community based on 16S rRNA sequencing. Raw sequence data were deposited in the NCBI Sequence Read Archive database. The SRA accession number is SRP158107.

### 2.8. Computational Analysis

Paired-end raw reads quality filtering were performed to obtain high-quality reads according to the Trimmomatic (V0.33) [23] quality controlled process. Paired-end clean reads were then merged using FLASH (V1.2.11) [24] to obtain raw tags. Raw tags were then assigned to each sample based on their unique barcode and primer using Mothur software (V1.35.1) [25] to get effective clean tags. Sequences analysis was performed using USEARCH software (V10) [26] pipelines. Sequences with ≥97% similarity were assigned to the same operational taxonomic unit (OTU). A representative sequence for each OTU was screened for further annotation. The resulting OTUs were used in all subsequent analyses. During the clustering, USEARCH can remove the chimera sequence and singleton OTU at the same time. The phylogenetic relationships of different OTUs were analyzed using KRONA software [27]. Phylogenetic relationship construction was conducted using FastTree software [28] for multiple sequence alignment and ggtree software for the visual display of the relative abundances of each OTU and the species annotation information. Based on the relative abundance of species at each classification level in OTU_table, vegan R package (V2.15.3) [29] was used to draw the histogram, heat map, and ternary plots. All indices of α-diversity and β-diversity analysis were conducted with QIIME (V1.9.1) [30] and displayed with R software (V2.15.3). α-diversity was analyzed through five indices including Observed species, Chao1, Shannon, Simpson, and dominance. All of these indices were calculated with QIIME (V1.9.1) and displayed with vegan R package (V2.15.3). Bray-curtis, weighted and unweighted unifrac β-diversity indexes were calculated by QIIME software. Principal Coordinate Analysis (PCoA) was performed to get principal coordinates and visualize from complex, multidimensional data. PCoA plot was analyzed by qiime2 and ggplot2 package in vegan R package (V2.15.3). Linear discriminant analysis effect size (LEfSe) analysis was used to find the biomarker of each group to display the extent of differences between (among) groups and whether the differences were significant. Cladogram plot was then built based on LEfSe analysis.

## 3. Results and Discussion

### 3.1. Characterization of Beads

**Figure 1:**
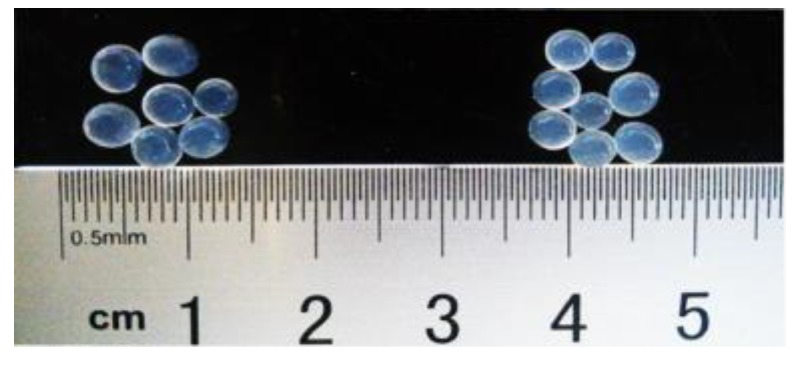
Morphology of both vacant beads (left) and HG10 beads (right) prepared from sodium alginate.

The average size of beads was shown to be 3–4 mm (Figure 1). No obvious size difference was observed between vacant and HG10 entrapping QQ beads. Successful entrapping of HG10 inside beads was observed by SEM (data not shown).

### 3.2. Impact of QQ Beads for Alleviating Biofouling on Filter Membranes

A flat-sheet membrane was submerged in each conical flask (i.e., a batch reactor) to simulate biofilm formation and microbial community structure on the membrane surface in an MBR.

**Figure 2:**
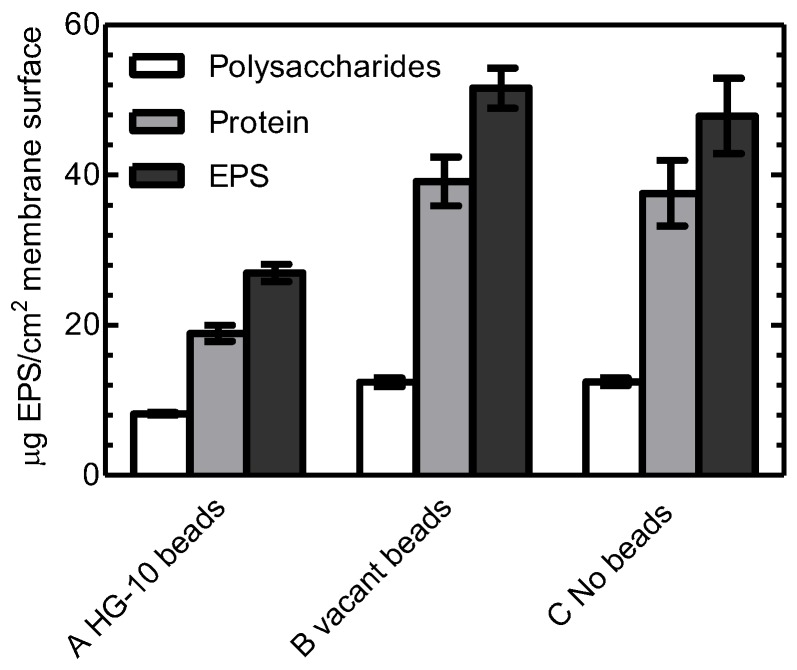
The amount of extracellular polymeric substances (EPS) on the surface of the polyvinyl difluoride (PVDF) membrane on the eighth day. Error bars represent standard deviations (*n* = 3).

Extracellular polymeric substances (EPS) are considered key components for the structure of biofilms. The amount of EPS (carbohydrates and proteins) in the biofilm on the eighth day were measured (Figure 2). In the presence of HG10 beads, on the eighth day, the amount of total carbohydrates and total proteins decreased by 34% and 50%, respectively, compared with those in the presence of vacant beads. Accordingly, total EPS levels dropped by 43% when HG10 beads were added. EPS reduction came mainly from the overall decrease in protein. The production of EPS is regulated by QS and helps biofilm formation. *Bacillus cereus* has been known for generating lactonase that can degrade AHL signal molecules [31,32,33]. Indeed, the *Bacillus cereus* HG10 strain showed substantial C6-HSL-degrading ability (data not shown). Thus, HG10 beads attenuated biofilm formation by reducing EPS excretion.

**Figure 3:**
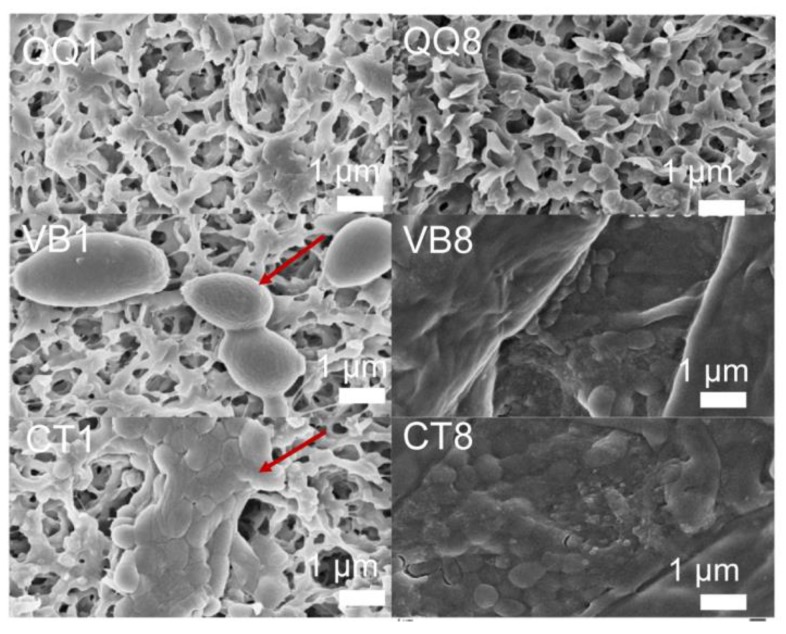
HG10 beads hindered biofouling on PVDF membranes. Scanning electron microscopy (SEM) images of PVDF membrane surfaces with quorum quenching (QQ) beads, vacant beads (VB), or the control without beads (CT): Left column, after 1 day incubation; right column, after 8 day incubation (magnification factor, 10,000×).

**Figure 4:**
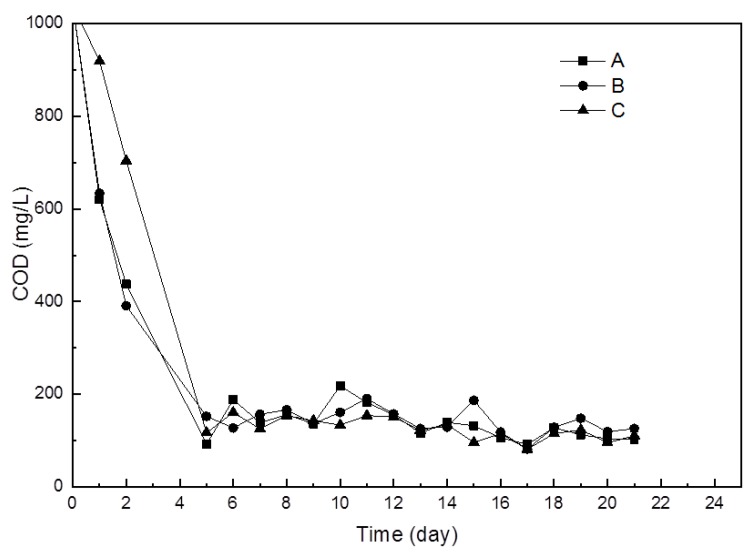
Chemical oxygen demand (COD) during the continuous 21-day incubation. Quorum quenching (QQ): in the presence of HG10 beads. VB: in the presence of vacant beads. CT: No beads were added.

Biofilms on the membrane surface in the batch bioreactors with and without beads were visualized on days 1 and 8 using scanning electron microscopy (SEM) (Figure 3). For the control (i.e., with no beads) (CT) group and vacant beads (VB) groups, biofilms formed substantially on the membrane surface on day 1 (Figure 3—CT1, VB1). In contrast, for the HG10 beads QQ (QQ) group, no biofilms were observed on day 1 (Figure 3—QQ1), suggesting that the initial attachment of microbes on the membrane surface was completely prevented. After another 7 days of continuous incubation, severe biofouling was observed in both the VB (Figure 3—VB8) and CT (Figure 3—CT8) groups. However, no obvious biofilms were observed for the QQ group (Figure 3—QQ8). Meanwhile, the COD in all three groups remained similar during the entire process (Figure 4), indicating that the addition of HG10 beads did not influence wastewater treatment efficiency but biofilm formation.

In summary, HG10-entrapped QQ beads effectively prevented or delayed the maturation of biofilms, proving that the QQ bacteria have anti-biofouling abilities under the experimental conditions in this study. Furthermore, HG10 QQ beads caused a reduction in EPS and thus inhibited biofilm formation without interfering with wastewater treatment efficiency.

### 3.3. Sequencing of Activated Sludge and Biofilm Samples

After running the three bioreactor groups in parallel for 20 days, a total of 601,341 high-quality sequencing reads were obtained from nine activated sludge and nine biofilm samples by 16S rRNA gene amplification. Low-quality sequences were removed, and the remaining effective reads were then clustered into 3613 operational taxonomic units (OTUs) at 97% sequence similarity. Rarefaction curves indicated that the sequencing depth was sufficient to detect all the genera within each sample. The results also indicated that the total number of sequences and OTUs in the QQ samples were less than those in the paralleled control samples.

### 3.4. Effect of QQ Beads on the Microbial Community Structure of Activated Sludge and Biofilm

**Figure 5:**
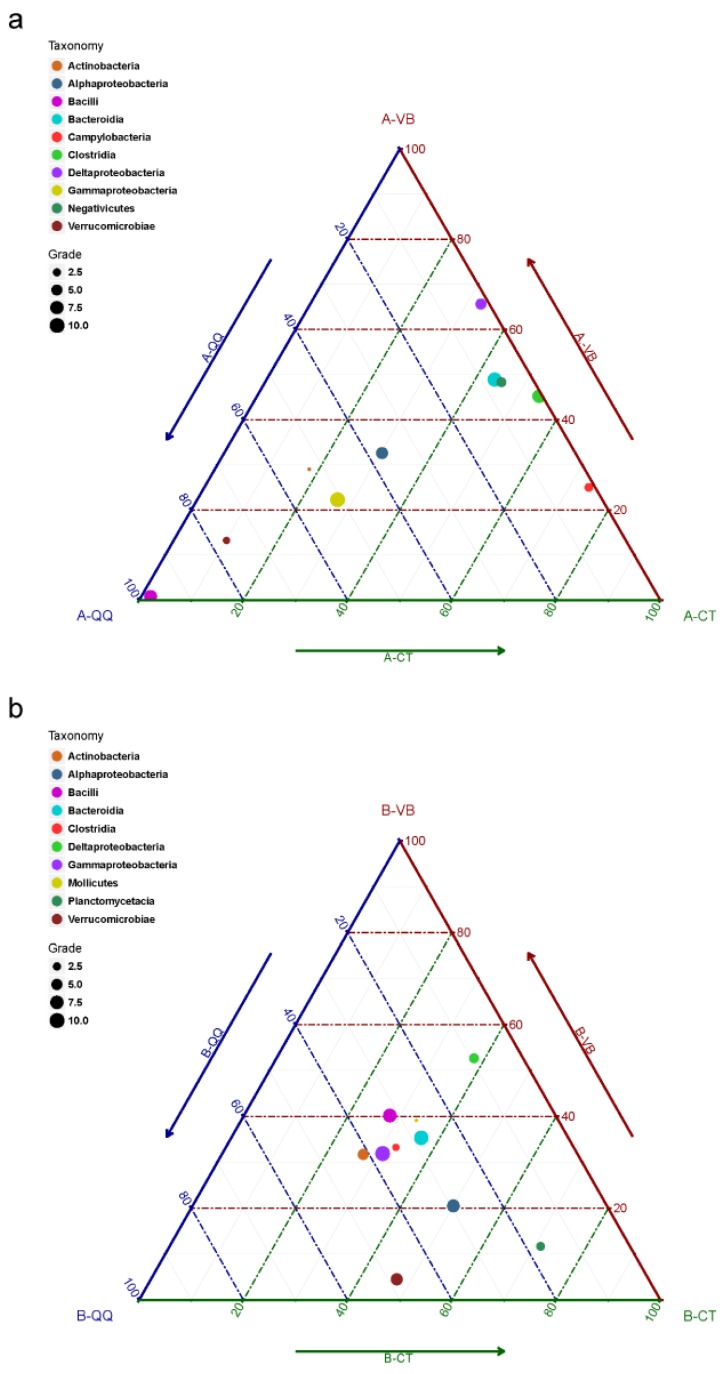
Ternary plot depicting the top 10 operational taxonomic units (OTUs) categorized under the class level with the highest relative abundance. (**a**) Activated sludge with quorum quenching (QQ) beads (A-QQ), vacant beads (A-VB), and control (A-CT), (**b**) biofilms formed on the membrane surface with QQ beads (B-QQ), vacant beads (B-VB), and control (B-CT). Grade represents the relative abundance (RA) of each bacteria, and a bigger grade number indicates a larger RA.

**Figure 6:**
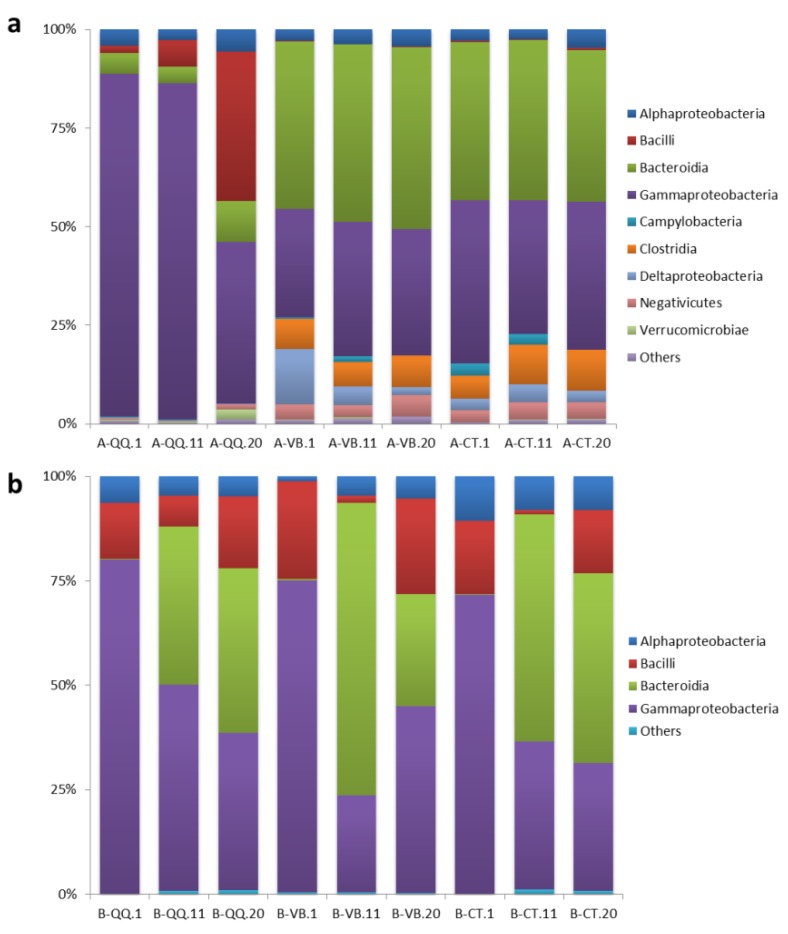
RA plot depicting bacteria grouped by the class taxonomic level with taxa abundance over 1%. (**a**) Activated sludge with quorum quenching (QQ) beads (A-QQ), vacant beads (A-VB), and control (A-CT), (**b**) biofilms formed on the membrane surface with QQ beads (B-QQ), vacant beads (B-VB), and control (B-CT). Numbers 1, 11, and 20 represent the sampling time in days from each bioreactor.

Although QQ beads have been demonstrated as an effective method to overcome biofouling in MBRs, little is known about the influence of QQ beads on the bacterial community, composition, and diversity. To investigate the effect of QQ beads on the microbial community structure of the activated sludge and the biofilms, bacterial community profiling analysis was conducted before and after the addition of QQ and vacant beads. In Figure 5, ternary plots are depicted for the top 10 OTUs categorized under the class level with the highest relative abundance (RA) in activated sludge (Figure 5a) and biofilm (Figure 5b), respectively. Figure 6 shows RA plots depicted for the bacteria grouped by class taxonomic level with taxa abundance over 1% in activated sludge (Figure 6a) and biofilm (Figure 6b).

As shown in Figure 5a and Figure 6a, the microbial community structure in activated sludge with QQ beads was greatly divergent from those in activated sludge with vacant or control beads. In contrast, in the biofilms, no significant difference in the microbial community structure was observed between the three groups (Figure 5b and Figure 6b). Taxonomic assignments at the class level for the top 10 OTUs with the highest relative abundance (RA) indicated that bacteria belonging to the class Gammaproteobacteria dominated the microbial community in activated sludge with QQ beads (A-QQ). Meanwhile, Gammaproteobacteria and Bacteroidia evenly dominated the microbial community in activated sludge with vacant beads (A-VB) and no beads (A-CT) (Figure 6a). However, the richness of Gammaproteobacteria in the A-QQ group was significantly higher than that of the controlled groups. Besides, more Bacilli and Verrucomicrobiae were observed in A-QQ group than A-VB and A-CT groups. 

In addition, the composition and abundance of microbes changed with incubation time (1, 11, and 20 days) both in activated sludge (Figure 6a) and in biofilm (Figure 6b). The RA of Gammaproteobacteria was 87% and 85% on the first and 11th days, respectively, in activated sludge with QQ beads (Figure 6a), which were approximately 40% higher than those with vacant beads and no beads at the same time. The RA of *Bacilli* increased with time of incubation in activated sludge with QQ beads from 1.8% (day 1) to 38% (day 20). However, it remained almost constant in the other two groups (with an average of 0.2%). In contrast, certain classes of bacteria, including Bacteroidia, Clostridia, Deltaproteobacteria, and Campylobacteria, were diminished in activated sludge with QQ beads.

However, no similar fluctuations in the microbial community were observed among the biofilms in all three groups (B-QQ, B-VB, and B-CT), as shown in Figure 5b and Figure 6b. Although the total biofilm mass in biofilms with QQ beads was the least among the three groups, the microbial community structure in the biofilm was similar. The dominant phyla in biofilms for all three groups were Proteobacteria and Bacteroidetes, which is consistent with previous observations [34]. As expected, the microbial richness was lower in the biofilm on the membrane surface than on the activated sludge at the same incubation time in all three groups, but the microbial diversity in the biofilm increased over time, indicating that QQ inhibited biofilm formation rather than killing microorganisms.

**Figure 7:**
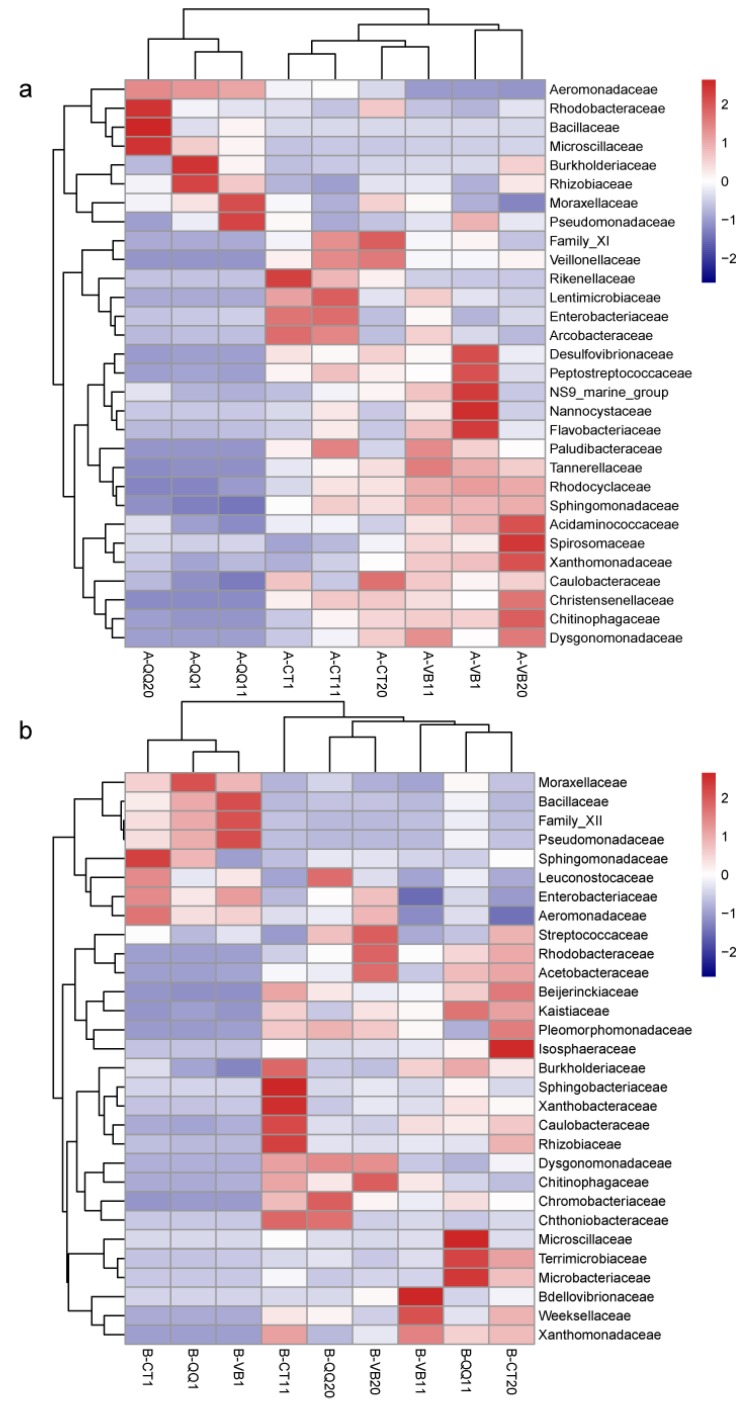
Heatmaps of the relative abundance for the top 30 bacteria grouped at the family level. (**a**) A-QQ, A-VB, and A-CT represent activated sludge with quorum quenching (QQ) beads, vacant beads, and no beads, respectively. (**b**) B-QQ, B-VB, and B-CT represent biofilms on membranes with QQ beads, vacant beads, and no beads, respectively. Numbers 1, 11, and 20 indicate the sampling time in days from each bioreactor. The color bar indicates intensity of each family in corresponding sample. Darker red indicated higher richness of certain family. Hierarchical cluster was shown on the left. Sample cluster (shown on the top) was created based on their community structure similarity.

The heatmaps drawn at the family level further illustrated a dissimilar composition and abundance of microbial structures between activated sludge with QQ beads and those in the other two groups (Figure 7a). At the family taxonomic level, Aeromonadaceae, Burkholderiaceae, Rhizobiaceae, Moraxellaceae, and Pseudomonadaceae were noticeably fortified with the addition of QQ beads. Meanwhile, more than 20 bacterial taxa, including Desulfovibrionaceae and Nannocystaceae, were reduced by the QQ beads. In contrast, in the biofilms, a similar pattern of microbial community structure and richness was observed between the three groups (Figure 7b).

**Figure 8:**
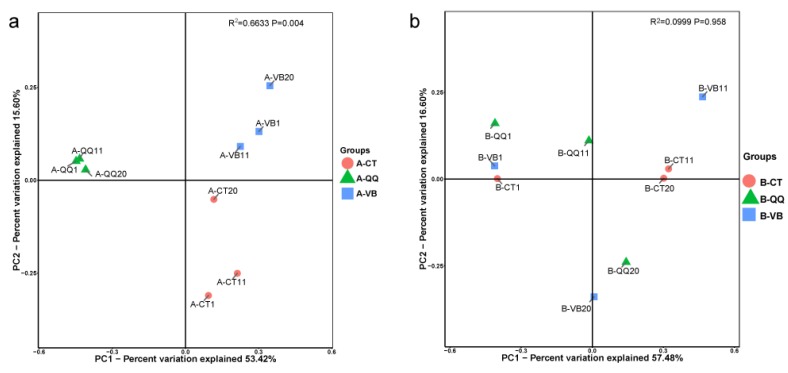
Principal coordinate analysis (PcoA) plot of Bray-Curtis distances. (**a**) The results demonstrated an obvious separation between samples from quorum quenching (QQ) beads (A-QQ, triangle), vacant beads (A-VB, square), and no beads (A-CT, circle); (**b**) Bray-Curtis distance metrics showed no obvious divergence between biofilms from QQ beads (B-QQ, triangle), vacant beads (B-VB, square), and no beads (B-CT, circle). Numbers 1, 11, and 20 indicate the sampling time in days.

**Figure 9:**
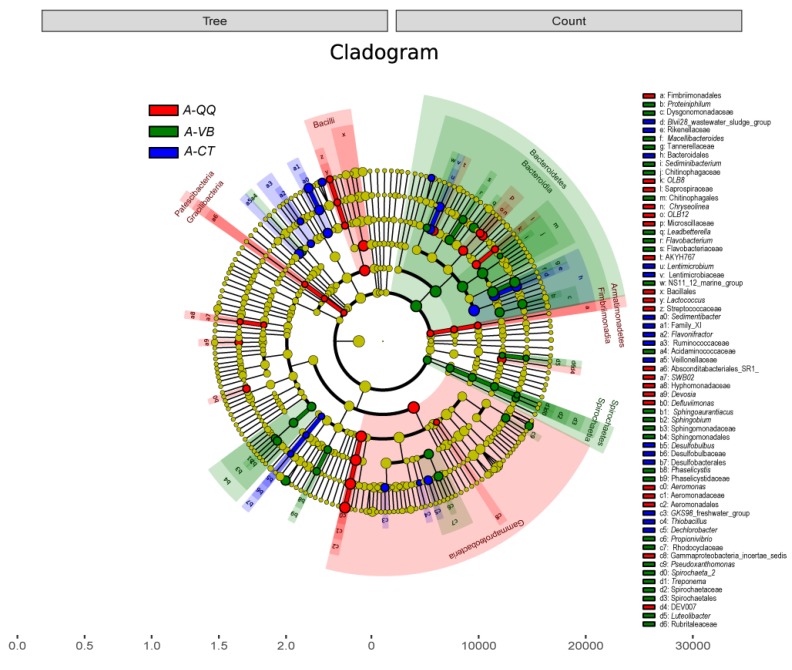
Plot of cladogram from linear discriminant analysis (LDA) effect size (LEfSe) analysis of activated sludge depicting the taxonomic levels represented by rings (from inside to outside: phyla to genera). Each circle is a member within each level. The size of each circle is proportional to RA. Taxa at each level showed a significant difference in abundance. Red-QQ beads (A-QQ); green-vacant beads (A-VB); blue-no beads control (A-CT).

To evaluate the difference in species complexity in both activated sludge and biofilms of the three groups, β-diversity (inter-sample diversity) was compared using Bray-Curtis distances. Principal coordinate analysis (PCoA) was performed (Figure 8). This analysis further indicated a clear disparity between activated sludge with QQ beads, vacant beads, and no beads (Figure 8a). No obvious separation was observed between the biofilm samples (Figure 8b). In addition, α-diversity analysis (intra-sample diversity) using the Shannon index revealed a decline of microbial community complexity in the activated sludge with QQ beads (Table S1). α-Diversity analysis using the observed species and Chao1 index revealed a decrease in microbial community richness in activated sludge with QQ beads. Meanwhile, the analysis using the Simpson or dominance indices indicated a more even distribution of microbiota in activated sludge with QQ beads than that with no beads. LDA effect size (LEfSe) analysis was also conducted to reveal the biomarker of each group at different levels. The phylogenetic tree cladogram shows an overall biomarker species distribution and relative abundance of three parallel samples in the phylogenetic tree (Figure 9).

**Figure 10:**
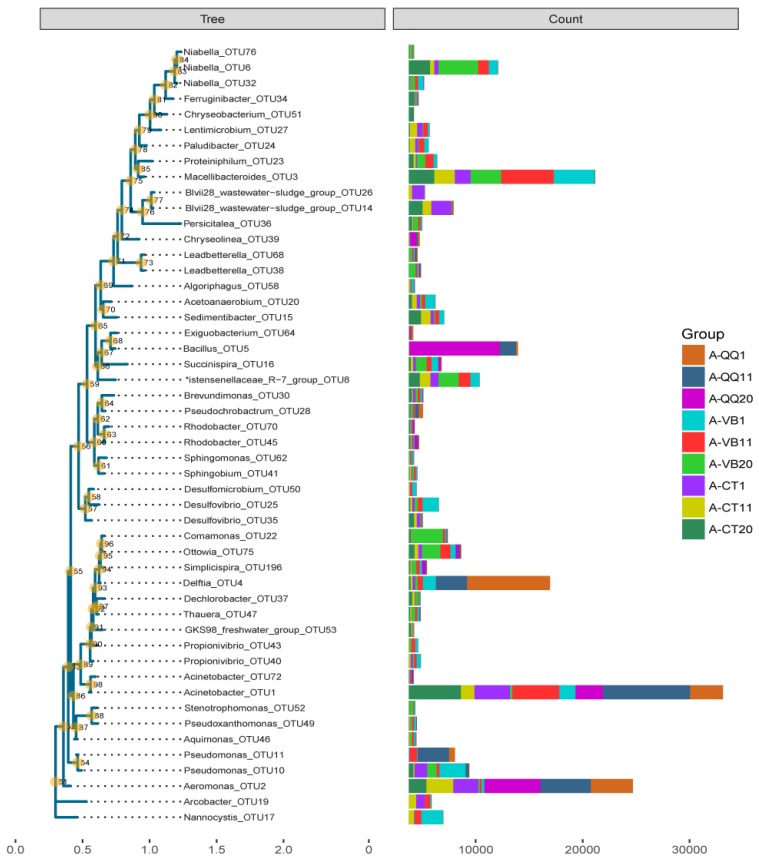
Plot of the phylogenetic tree showing the OTUs with the RA of the top 50 to the taxa level of genus in activated sludge with quorum quenching (QQ) beads (A-QQ), vacant beads (A-VB), and no beads (A-CT). The numbers at the branches were confidence values. The name after each branch indicates representative OTU annotations. The histogram to the left side represents the RA of each genus, and different colors were used to distinguish different samples.

To further uncover the difference in dominant species between different groups, the OTU representative sequence was analyzed with the relative abundance of the first 50 and annotated to the level of genus for the activated sludge in three groups (A-QQ, A-VB, and A-CT) (Figure 10). At the genus level, several bacteria with the highest count number stood out. QQ beads dramatically improved the growth of *Acinetobacter*_OTU1, *Aeromonas*_OTU2, *Delftia*_OTU4, *Bacillus*_OTU5, and *Pseudomonas*_OTU11 and hindered the growth of *Macellibacteroides*_OTU3, *Niabella*_OTU6 and OTU32, Christensenellaceae_R-7_group_OTU8, *Ottowia*_OTU75, *Pseudomonas*_OTU10, *Nannocystis*_OTU17, *Desulfovibrio*_OTU25, *Sedimentibacter*_OTU15, *Acetoanaerobium*_OTU20, and Blvii28_wastewater-sludge_group_OTU14/OTU26.

It is noteworthy that OTU5 was successfully defined to the level of species as *Bacillus cereus,* which is the same bacterial species that was embedded into the beads. The highest count of this species in the control group (i.e., with no beads) was only 17, whereas in the QQ group (i.e., with QQ beads), the number reached up to 1523 on the 11th day. The count of *Delftia* also increased dramatically with QQ beads. Interestingly, several species of the *Delftia* genera have been reported as having AHL-degrading activity and can inhibit QS. Singh et al. demonstrated that *Delftia tsuruhatensis* attenuates biofilm formation of *Pseudomonas aeruginosa* [35]. Others have reported that *Delftia* sp. interferes with QS by producing AHL acylase and can use AHL as the sole carbon source [36]. *Delftia acidovorans* has been reported to inhibit QS by modifying C6- to C8-HSL [37]. Concurrently, we also isolated *Delftia tsuruhatensis* strain NBRC 16741 and *Bacillus cereus* HG10 from the same activated sludge sample. This strain also showed high AHL-degrading activity (data not shown). *Aeromonas*_OTU2 and *Pseudomonas*_OTU11 were also successfully defined to the species level as *Aeromonas hydrophila* subsp. *hydrophila* and *Pseudomonas plecoglossicida*, respectively. However, limited reports have been found about these two strains.

Among the genera that were downregulated, Blvii28_wastewater-sludge_group has been commonly found in wastewater or wastewater treatment systems [38,39]. Researchers have indicated that this genus functions in the initial attachment during the biofilm forming process. In addition, the genus *Pseudomonas*_OTU10 was annotated to the level of species as *Pseudomonas otitidis*. This strain has been reported to have biofilm forming ability and has been applied to degrade oil in biofilm-based reactors [40].

Although several studies have reported the effectiveness of QQ in biofouling control, limited information is available on the influence of QQ bacteria on microbial community composition, diversity, and richness. Here, we demonstrated a significant influence of QQ beads on the microbial community structure in activated sludge while reducing biofouling on the membrane surface. These results raised the possibility that QQ bacteria, by interfering with cell-cell communication between biofilm-forming bacteria, might trigger other native quorum-quenching bacteria to flourish in the community and initiate quorum quenching in a synergistic manner. However, the detailed synergism or antagonism relationship between those microbes and their detailed mechanisms are intriguing and remain to be elucidated.

## 4. Conclusions

Immobilized indigenous *Bacillus cereus* effectively reduced biofouling on a membrane in a bioreactor. Remarkably, the bacterial community profiling data revealed a broad impact of QQ beads on the microbial distribution and abundance in activated sludge in a bioreactor. Clear separation of bacterial structure was observed at all genera and phyla taxonomic ranks. Certain OTUs clustered to the genus level, including *Acinetobacter*, *Aeromonas*, *Delftia*, *Bacillus*, and *Pseudomonas*, were strikingly enriched by QQ beads in activated sludge. Meanwhile, more than 12 other OTUs in activated sludge were depleted. Whereas biofilm formation on membranes with QQ beads was delayed, microbial structure divergence in biofilms was not as dramatic as that observed in the activated sludge in the bioreactor. Our results provide new insight into how QQ bacteria battle biofilm-forming microbes while affecting the entire bacterial community. The mechanisms turned out to be more complex than expected. Our observation implies the possibility that embedded QQ bacteria could trigger certain native microbial taxa to flourish and inhibit biofouling in a synergistic manner by interfering with bacterial communication. Thus, it might provide helpful information for future QQ-MBR design.

## Supplementary Materials

The following are available online at https://www.mdpi.com/1660-4601/16/19/3777/s1, Table S1: α-Diversity index of activated sludge samples with QQ beads (A-QQ), vacant beads (A-VB), and control (A-CT) and biofilm samples on filter membrane with QQ beads (B-QQ), vacant beads (B-VB), and control (B-CT) on the first, 11th, and 20th day.

## References

[1] Krzeminski P., Leverette L., Malamis S., Katsou E. (2017). Membrane bioreactors—A review on recent developments in energy reduction, fouling control, novel configurations, LCA and market prospects. J. Membr. Sci..

[2] Meng F., Zhang S., Oh Y., Zhou Z., Shin H.-S., Chae S.-R. (2017). Fouling in membrane bioreactors: An updated review. Water Res..

[3] Zhang X., Wang Z., Chen M., Ma J., Chen S., Wu Z. (2017). Membrane biofouling control using polyvinylidene fluoride membrane blended with quaternary ammonium compound assembled on carbon material. J. Membr. Sci..

[4] Amouamouha M., Badalians Gholikandi G. (2017). Characterization and Antibiofouling Performance Investigation of Hydrophobic Silver Nanocomposite Membranes: A Comparative Study. Membranes.

[5] Chen M., Zhang X., Wang Z., Wang L., Wu Z. (2017). QAC modified PVDF membranes: Antibiofouling performance, mechanisms, and effects on microbial communities in an MBR treating municipal wastewater. Water Res..

[6] Deng L., Guo W., Ngo H.H., Zhang H., Wang J., Li J., Xia S., Wu Y. (2016). Biofouling and control approaches in membrane bioreactors. Bioresour. Technol..

[7] Milenkovic J., Hrenovic J., Goic-Barisic I., Tomic M., Djonlagic J., Rajic N. (2014). Synergistic anti-biofouling effect of Ag-exchanged zeolite and D-Tyrosine on PVC composite against the clinical isolate of Acinetobacter baumannii. Biofouling.

[8] Bouayed N., Dietrich N., Lafforgue C., Lee C.H., Guigui C. (2016). Process-Oriented Review of Bacterial Quorum Quenching for Membrane Biofouling Mitigation in Membrane Bioreactors (MBRs). Membranes.

[9] Cheong W.S., Kim S.R., Oh H.S., Lee S.H., Yeon K.M., Lee C.H., Lee J.K. (2014). Design of quorum quenching microbial vessel to enhance cell viability for biofouling control in membrane bioreactor. J. Microbiol. Biotechnol..

[10] Kim S.R., Oh H.S., Jo S.J., Yeon K.M., Lee C.H., Lim D.J., Lee C.H., Lee J.K. (2013). Biofouling control with bead-entrapped quorum quenching bacteria in membrane bioreactors: physical and biological effects. Environ. Sci. Technol..

[11] Nguyen T., Roddick F.A., Fan L. (2012). Biofouling of Water Treatment Membranes: A Review of the Underlying Causes, Monitoring Techniques and Control Measures. Membranes.

[12] Watnick P., Kolter R. (2000). Biofilm, City of Microbes. J. Bacteriol..

[13] Morgan-Sagastume F., Boon N., Dobbelaere S., Defoirdt T., Verstraete W. (2005). Production of acylated homoserine lactones by Aeromonas and Pseudomonas strains isolated from municipal activated sludge. Can. J. Microbiol..

[14] Chong G., Kimyon O., Rice S.A., Kjelleberg S., Manefield M. (2012). The presence and role of bacterial quorum sensing in activated sludge. Microbiol. Biotechnol..

[15] Yeon K.M., Cheong W.S., Oh H.S., Lee W.N., Hwang B.K., Lee C.H., Beyenal H., Lewandowski Z. (2009). Quorum sensing: a new biofouling control paradigm in a membrane bioreactor for advanced wastewater treatment. Environ. Sci. Technol..

[16] Siddiqui M.F., Sakinah M., Singh L., Zularisam A.W. (2012). Targeting N-acyl-homoserine-lactones to mitigate membrane biofouling based on quorum sensing using a biofouling reducer. J. Biotechnol..

[17] Lee K., Lee S., Lee S.H., Kim S.R., Oh H.S., Park P.K., Choo K.H., Kim Y.W., Lee J.K., Lee C.H. (2016). Fungal Quorum Quenching: A Paradigm Shift for Energy Savings in Membrane Bioreactor (MBR) for Wastewater Treatment. Environ. Sci. Technol..

[18] Ochiai S., Morohoshi T., Kurabeishi A., Shinozaki M., Fujita H., Sawada I., Ikeda T. (2013). Production and degradation of N-acylhomoserine lactone quorum sensing signal molecules in bacteria isolated from activated sludge. Biosci. Biotechnol. Biochem..

[19] ZHAO C., WANG W.Z., XU Q.Y. (2016). Isolation of Quorum Quenching Bacteria and Their Function for Controlling Membrane Biofouling. Environ. Sci..

[20] APHA (2005). APHA Standard Methods for Water and Wastewater Examination.

[21] DuBois M., Gilles K.A., Hamilton J.K., Rebers P.A., Smith F. (1956). Colorimetric Method for Determination of Sugars and Related Substances. Anal. Chem..

[22] Judd S. (2011). The MBR Book.

[23] Bolger A.M., Lohse M., Usadel B. (2014). Trimmomatic: a flexible trimmer for Illumina sequence data. Bioinformatics.

[24] Magoc T., Salzberg S.L. (2011). FLASH: fast length adjustment of short reads to improve genome assemblies. Bioinformatics.

[25] Schloss P.D., Westcott S.L., Ryabin T., Hall J.R., Hartmann M., Hollister E.B., Lesniewski R.A., Oakley B.B., Parks D.H., Robinson C.J. (2009). Introducing mothur: Open-Source, Platform-Independent, Community-Supported Software for Describing and Comparing Microbial Communities. Appl. Environ. Microbiol..

[26] Edgar R.C. (2010). Search and clustering orders of magnitude faster than BLAST. Bioinformatics.

[27] Ondov B.D., Bergman N.H., Phillippy A.M. (2011). Interactive metagenomic visualization in a Web browser. BMC Bioinformatics.

[28] Price M.N., Dehal P.S., Arkin A.P. (2010). FastTree 2--approximately maximum-likelihood trees for large alignments. PloS ONE.

[29] Oksanen J., Blanchet F.G., Kindt R., Legendre P., Minchin P.R., O’Hara R.B., Simpson G.L., Solymos P., Stevenes M.H.H., Wagner H.H. (2013). Vegan: Community Ecology Package. R version 2.

[30] Caporaso J.G., Kuczynski J., Stombaugh J., Bittinger K., Bushman F.D., Costello E.K., Fierer N., Pena A.G., Goodrich J.K., Gordon J.I. (2010). QIIME allows analysis of high-throughput community sequencing data. Nat. Methods.

[31] Medina-Martínez M.S., Uyttendaele M., Rajkovic A., Nadal P., Debevere J. (2007). Degradation of N-Acyl-l-Homoserine Lactones by Bacillus cereus in Culture Media and Pork Extract. Appl. Environ. Microbiol..

[32] Yavuztürk Gül B., Imer D.Y., Park P.-K., Koyuncu I. (2017). Evaluation of a novel anti-biofouling microorganism (Bacillus sp. T5) for control of membrane biofouling and its effect on bacterial community structure in membrane bioreactors. Water Sci. Technol..

[33] Zamani M., Behboudi K., Ahmadzadeh M. (2013). Quorum quenching by Bacillus cereus U92: a double-edged sword in biological control of plant diseases. Biocontrol Sci. Technol..

[34] Jo S.J., Kwon H., Jeong S.Y., Lee S.H., Oh H.S., Yi T., Lee C.H., Kim T.G. (2016). Effects of Quorum Quenching on the Microbial Community of Biofilm in an Anoxic/Oxic MBR for Wastewater Treatment. J. Microbiol. Biotechn..

[35] Singh V.K., Mishra A., Jha B. (2017). Anti-quorum Sensing and Anti-biofilm Activity of Delftia tsuruhatensis Extract by Attenuating the Quorum Sensing-Controlled Virulence Factor Production in Pseudomonas aeruginosa. Front. Cell. Infect. Microbiol..

[36] Maisuria V.B., Nerurkar A.S. (2015). Interference of Quorum Sensing by Delftia sp. VM4 Depends on the Activity of a Novel N-Acylhomoserine Lactone-Acylase. PloS ONE.

[37] Uroz S., Dessaux Y., Oger P. (2009). Quorum sensing and quorum quenching: the yin and yang of bacterial communication. Chem. Bio. Chem..

[38] Lefevre E., Redfern L., Cooper E.M., Stapleton H.M., Gunsch C.K. (2019). Acetate promotes microbial reductive debromination of tetrabromobisphenol A during the startup phase of anaerobic wastewater sludge bioreactors. Sci. Total Environ..

[39] Zhang L., Zhang S., Lv X., Qiu Z., Zhang Z., Yan L. (2018). Dissolved organic matter release in overlying water and bacterial community shifts in biofilm during the decomposition of Myriophyllum verticillatum. Sci. Total Environ..

[40] Dasgupta D., Ghosh R., Sengupta T.K. (2013). Biofilm-mediated enhanced crude oil degradation by newly isolated pseudomonas species. ISRN Biotechnol..

