# Supplementary material for: Anti-Biofouling Performance of an Immobilized Indigenous Quorum Quenching Bacterium Bacillus cereus HG10 and Its Influence on the Microbial Community in a Bioreactor
Source: Int J Environ Res Public Health. 2019 Oct 8;16(19):3777. doi: 10.3390/ijerph16193777 (PMC6802356; doi:10.3390/ijerph16193777)
Supplement: Supplementary file 1 [file ijerph-16-03777-s001.pdf]

Supplementary Materials

Table S1. α-Diversity index of activated sludge samples with QQ beads (A-QQ), vacant beads (A-VB) and control (A-CT) and biofilm samples on filter membrane with QQ beads (B-QQ), vacant beads (B-VB) and control (B-CT) on the 1st, 11th and 20th day.

Sample ID	PD_whole_tree	chao1	dominance	observed_species	shannon	simpson
A-QQ1	20	271.800	0.252	216	3.022	0.748
A-QQ11	18	239.845	0.194	199	3.233	0.806
A-QQ20	21	295.105	0.208	232	3.514	0.792
A-VB1	25	312.792	0.046	267	5.380	0.954
A-VB11	24	308.103	0.060	264	5.309	0.940
A-VB20	23	313.284	0.056	259	5.196	0.944
A-CT1	19	272.108	0.065	224	4.813	0.935
A-CT11	23	319.595	0.045	246	5.329	0.955
A-CT20	23	292.582	0.064	249	5.117	0.936
B-QQ1	12	153.567	0.194	118	2.939	0.806
B-QQ11	15	178.702	0.099	153	4.113	0.901
B-QQ20	12	180.769	0.120	135	3.796	0.880
B-VB1	13	174.693	0.216	139	2.829	0.784
B-VB11	14	167.299	0.300	137	2.978	0.700
B-VB20	14	200.668	0.184	149	3.294	0.816
B-CT1	13	132.029	0.226	108	2.872	0.774
B-CT11	15	183.799	0.096	150	4.179	0.904
B-CT20	16	181.478	0.118	152	4.008	0.882
